# Bridging the Gap Between Fluid Biomarkers for Alzheimer’s Disease, Model Systems, and Patients

**DOI:** 10.3389/fnagi.2020.00272

**Published:** 2020-09-02

**Authors:** Christiana Bjorkli, Axel Sandvig, Ioanna Sandvig

**Affiliations:** ^1^Sandvig Group, Department of Neuromedicine and Movement Science, Faculty of Medicine and Health Sciences, Norwegian University of Science and Technology, Trondheim, Norway; ^2^Institute of Neuromedicine and Movement Science, Department of Neurology, St. Olavs Hospital, Trondheim, Norway; ^3^Department of Pharmacology and Clinical Neurosciences, Division of Neuro, Head, and Neck, University Hospital of Umeå, Umeå, Sweden

**Keywords:** Alzheimer’s disease, translational research, biomarkers, cerebrospinal fluid, screening tools

## Abstract

Alzheimer’s disease (AD) is a debilitating neurodegenerative disease characterized by the accumulation of two proteins in fibrillar form: amyloid-β (Aβ) and tau. Despite decades of intensive research, we cannot yet pinpoint the exact cause of the disease or unequivocally determine the exact mechanism(s) underlying its progression. This confounds early diagnosis and treatment of the disease. Cerebrospinal fluid (CSF) biomarkers, which can reveal ongoing biochemical changes in the brain, can help monitor developing AD pathology prior to clinical diagnosis. Here we review preclinical and clinical investigations of commonly used biomarkers in animals and patients with AD, which can bridge translation from model systems into the clinic. The core AD biomarkers have been found to translate well across species, whereas biomarkers of neuroinflammation translate to a lesser extent. Nevertheless, there is no absolute equivalence between biomarkers in human AD patients and those examined in preclinical models in terms of revealing key pathological hallmarks of the disease. In this review, we provide an overview of current but also novel AD biomarkers and how they relate to key constituents of the pathological cascade, highlighting confounding factors and pitfalls in interpretation, and also provide recommendations for standardized procedures during sample collection to enhance the translational validity of preclinical AD models.

## Introduction

Due to an increasingly elderly population, patients with Alzheimer’s disease (AD) constitute a growing public health problem, thus developing methods for early diagnosis of the disease will become pertinent as there of yet exists no cure. The disease typically manifests through a progressive decline in cognitive and behavioral functions that severely impact the ability of AD patients to independently perform daily tasks. As a result, the associated socioeconomic cost and burden to the healthcare system are very high, with annual healthcare expenditure exceeding billions of dollars. Based on the early findings by Alzheimer et al. (1995), we now know that the neuropathological hallmarks of AD include intracellular neurofibrillary tangles (NFTs) composed of misfolded tau protein, and extracellular amyloid plaques comprising aggregated amyloid-β (Aβ). The pathological protein accumulation in AD follows a predictable spatiotemporal pattern where certain areas become affected before others, including the entorhinal cortex (EC) and the hippocampus (Serrano-Pozo et al., 2011). In late stage AD, up to 90% of cells are lost in EC layer II (Gomez-Isla et al., 1996). The initial Aβ deposits present as plaques in the temporal neocortex, before progressing to the EC and the hippocampus (Box 1; Thal et al., 2002). Meanwhile, initial tangle formation begins in the most lateral portions of EC layer II, followed by the hippocampus, before appearing in areas of the neocortex (Box 1; Braak and Braak, 1991). The anatomical and temporal progression of Aβ and tau pathology, and subsequently neurodegeneration, has led to the postulation that Aβ acts as an initiator of the disease progression that results in tau-mediated neurodegeneration (Freudenberg-Hua et al., 2018).

**BOX 1:**
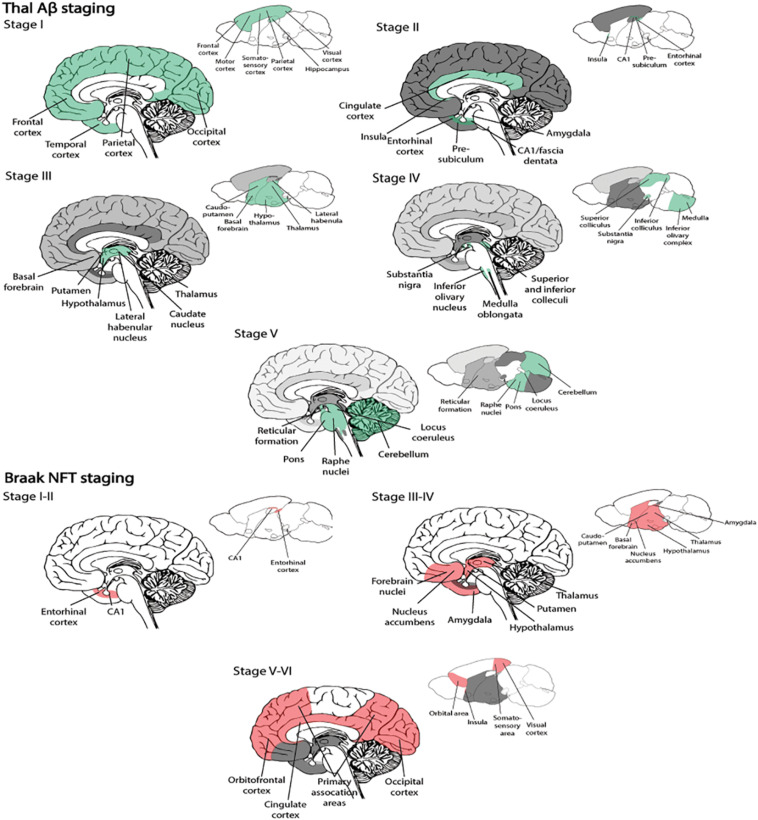
Spatiotemporal pattern of Aβ and NFT deposition during the AD disease cascade in the human and mouse brain. Stages of Aβ deposition in the AD brain (Gomez-Isla et al., 1996). Stage I is characterized by exclusively neocortical Aβ deposits (neocortex: green) (Gomez-Isla et al., 1996). This includes Aβ deposits in frontal, temporal, parietal, and occipital cortices (Gomez-Isla et al., 1996). Stage II shows additional allocortical Aβ deposits (green) in entorhinal cortex, CA1, cingulate cortex, amygdala, presubiculum, and the fascia dentata (Gomez-Isla et al., 1996). In stage III, there are additional Aβ deposits in diencephalic nuclei and striatum (green) including thalamus, hypothalamus, the basal forebrain, caudate nucleus, putamen, claustrum, the lateral habenular nucleus, and white matter (Gomez-Isla et al., 1996). In stage IV there are Aβ deposits in distinct brainstem nuclei (substantia nigra, superior and inferior colliculi, inferior olivary nucleus, intermediate reticular zone, central gray of the midbrain, CA4, and the red nucleus; green) (Gomez-Isla et al., 1996). In stage V there are Aβ deposits in the cerebellum and additional brainstem nuclei (pons, locus coeruleus, reticular formation, raphe nuclei, parabrachial nuclei, and the dorsal tegmental nucleus; green) (Gomez-Isla et al., 1996). Stages of NFT deposition in the AD brain (Thal et al., 2002). Stages I-II show alterations which are confined to the superficial entorhinal cellular layer (pre-α; layer II/layer IIa) (Thal et al., 2002; Freudenberg-Hua et al., 2018). The next stage is an aggravation of stage I (Thal et al., 2002). Stages III-IV lead to severe involvement of the entorhinal and transentorhinal layer pre-α (pink) (Thal et al., 2002). Stage IV is characterized by layer pre-α, pri-α (layer V) (Freudenberg-Hua et al., 2018), and pre-β (layer III; layer IIb) (Thal et al., 2002; Freudenberg-Hua et al., 2018) involvement. CA1, the basolateral nuclei of the amygdala, the reuniens nucleus, the antero-dorsal thalamic nucleus, putamen, and nucleus accumbens are densely filled with NFTs (Thal et al., 2002). Stages V-VI are marked by isocortical destruction (pink) (Thal et al., 2002). In stage V, the deep layer pri-α is severely involved. Layers pre-β and pre-γ (layer III) (Freudenberg-Hua et al., 2018) are also affected (Thal et al., 2002). Virtually all components of the hippocampal formation are involved, and the isocortex is severely affected (Thal et al., 2002). By stage VI, the subcortical nuclei show a much more pronounced involvement (Thal et al., 2002), and considerable nerve cell loss is seen in layers pre-α and pri-α (Thal et al., 2002). Grayscale represents the recency of involved regions for each stage of neuropathology. Human brain regions adapted from Jürgen et al. (2016); mouse brain regions adapted from Allen brain atlas (Sunkin et al., 2013).

**FIGURE 1:**
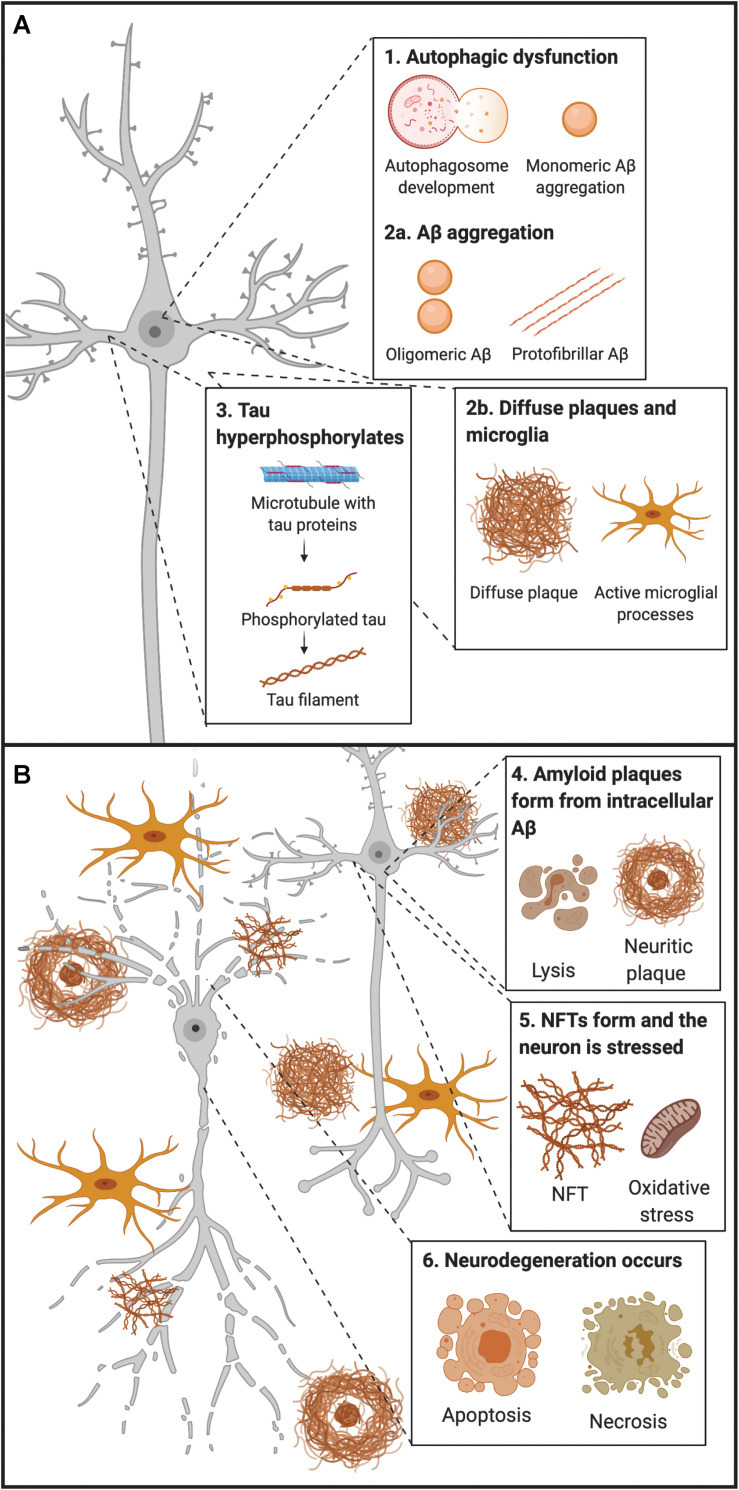
Early and late timepoints of neuropathological development in AD. **(A)** Early molecular abnormalities leading to the development of neuropathological hallmarks in AD. First, disruption of endocytic and autophagic systems may lead to, or simultaneously become disrupted, as monomeric Aβ aggregates. Following, monomeric Aβ aggregates into oligomeric and protofibrillar forms. At the same time, diffuse amyloid plaques may be present extracellularly and begin to sequester Aβ from synapses, leading to microglial activation (and other inflammatory responses). Next, tau proteins become hyperphosphorylated and move from the axon to the somatodendritic compartment of the neuron. **(B)** Late molecular abnormalities in AD. While diffuse plaques are present extracellularly and may become neuritic by sequestering Aβ peptides from the synapse, a lysis event of the neuron occurs, and intracellular protofibrillar Aβ is deposited into the extracellular space leading to the development of neuritic plaques (composed of fibrillar Aβ). Other amyloid plaques in close vicinity may sequester Aβ peptides between each other, leading to the increased accumulation of neuritic plaques. Next, hyperphosphorylated tau from paired helical filaments form NFTs which results in excitotoxicity and oxidative stress. Ultimately neurodegeneration of the neuron occurs, primarily by necrosis, but apoptosis may also occur from some intracellular processes. Aβ, amyloid-β; NFT, neurofibrillary tangle.

Many variants of the amyloid cascade hypothesis have been proposed over the years; and this hypothesis argues that the deposition of Aβ is the initial and causative step for developing AD (Hardy and Higgins, 1992). According to this hypothesis, Aβ deposition causes disruption of calcium homeostasis in cells, resulting in molecular lesions, NFTs, oxidative stress, inflammation, excitotoxicity, and eventually cell death. The main counter argument for this hypothesis has been that amyloid plaque burden has a low correlation with the severity of clinical symptoms of AD, unlike that of NFTs and neurodegeneration (Terry et al., 1991; Arriagada et al., 1992). In line with this, amyloid plaque deposition commonly plateaus with time, despite declining cognition in AD (Engler et al., 2006). Therefore, the majority in the AD research field now focus on soluble, intracellular Aβ oligomers as a possible initiator of the development of the disease (Figure 1).

Amyloid-β can exist in multiple assembly forms, ranging from monomeric to oligomeric and fibrillar forms (Figure 1). As a monomer, Aβ does not seem to be toxic, whereas oligomeric or fibrillar forms have been found to be potent blockers of long-term potentiation (LaFerla et al., 2007). Research suggests that levels of soluble Aβ oligomers are better correlated with disease severity than amyloid plaques mainly consisting of insoluble Aβ fibrillar species (Arriagada et al., 1992; Lue et al., 1999; McLean et al., 1999; Naslund et al., 2000; Haass and Selkoe, 2007). When produced intracellularly, Aβ oligomers expose flexible hydrophobic surfaces that might contribute to trapping vital proteins and, in this way, they can subtly damage and predispose vulnerable neurons to the formation of intracellular tau aggregates (Campioni et al., 2010). Thus, tau pathology in AD appears to be a downstream, effect of the presence of Aβ oligomers (Figure 1). In line with this, a link has been made between increased amounts of intracellular Aβ and neurodegeneration, while clearing of intracellular Aβ has been shown to revert AD-related memory deficits in animals modeling AD (Billings et al., 2005). An explanation for the weak correlation between cognitive decline and plaque load could be that insoluble fibrillar Aβ species might serve as reservoirs for smaller oligomeric Aβ, thus sequestering these away from neurons (Mucke and Selkoe, 2012; Figure 1).

The manner in which pathology progresses in model systems and human patients with AD has mostly been investigated separately resulting in little or poor translational value. As such, the translational aspect of staging the AD molecular disease cascade between preclinical models and human AD patients has remained inadequate. Despite intense investigation into disease cause and mechanisms of neurodegeneration, there is currently no cure or unequivocal evidence as to the exact nature of its underlying cause. Still, there seems to be consensus as to the fact that the success of treatments is primarily contingent on whether they can target disease-related pathology at early onset. This suggests that we are urgently in need of better tools for early onset diagnosis, before evolving pathology severely affects brain function, as well as better tools for monitoring pathological progression.

This effectively means, to translate discoveries made in preclinical models to the clinic, we must bridge the gap between model systems and patients with AD by improving the robustness and predictive validity of screening tools. For instance, the current dominant view is that Aβ42 accumulates extracellularly first, and thereby leads to the formation of amyloid plaques. However, several studies of brain tissue from animal models and human patients have begun to challenge this notion. In this paper, we explore potential early screening tools for the diagnosis of AD and also provide links between the extensive research done in preclinical models to human clinical applications. Specifically, we review how screening in AD patients can become more precise by the use of novel cerebrospinal fluid (CSF) biomarkers and by following recommendations for standardized procedures during CSF sample collections. We also focus on how intracellular events of Aβ and tau aggregation eventually lead to extracellular deposition and the presence of neuropathological hallmarks, and how current tools can predict, diagnose and potentially treat models and patients at various timepoints of the disease.

## AD Biomarkers – Type and Definition

When defining an AD biomarker, many agree that it is a measurable indicator within a patient that can help to test and monitor the progress of pathology (Hane et al., 2017). The ideal fluid biomarker for AD would be consistent, reproducible, non-invasive, simple to measure, inexpensive, and easy to implement into the clinic and the primary care setting (Davies et al., 1998; Wang et al., 2012; Bjerke and Engelborghs, 2018; Molinuevo et al., 2018). Such biomarkers should be able to identify the clinical disease stage of the patient and also monitor treatment effects. Conventionally, patients with overt dementia are diagnosed with around 85% specificity (but at much lower rates in patients with early stage AD), but the ideal biomarker should exceed this rate (Davies et al., 1998). There is thus an urgent need for a specific marker for early detection in these patients. Various biomarkers that can detect early AD in both preclinical models and patients have been proposed. For instance, it would be preferable to have a biomarker that can detect intracellular events prior to the deposition of amyloid plaques and NFTs. In line with this, when diagnosing patients based on physical symptoms, reduced memory recall manifests in many diseases other than AD (Hane et al., 2017), highlighting the need for preclinical markers specific to AD.

**FIGURE 2:**
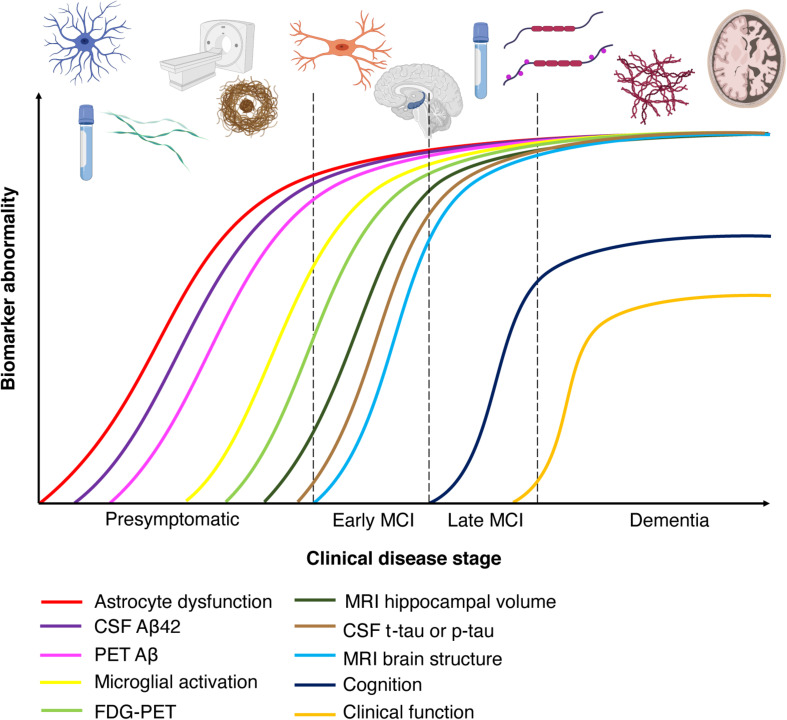
Chronobiological biomarkers to Alzheimer’s disease clinical stage. This disease model displays that biomarkers become abnormal in a temporally ordered manner as the disease progresses (Jack et al., 2010). Amyloid plaque biomarkers are dynamic early in the disease, prior to the appearance of clinical symptoms, and have largely reached a plateau by the time clinical symptoms appear (Jack et al., 2010). Biomarkers of neuronal injury, dysfunction, and degeneration are dynamic later in the disease and correlate with clinical symptom severity. MRI is the last biomarker to become aberrant. None of the biomarkers are static, and rates of change in each biomarker vary over time and follow a non-linear time-course, which is hypothesized to be sigmoid shaped (Jack et al., 2010). A sigmoid shape as a function of time implies that the maximum effect of each biomarker varies over the course of disease progression (Jack et al., 2010). Figure adapted with permission from Leclerc and Abulrob (2013). MCI, mild cognitive impairment; CSF, cerebrospinal fluid; Aβ, amyloid-β; PET, positron emission tomography; FDG, fluorine-based tracers; MRI, magnetic resonance imaging; t-tau, total tau; p-tau, phosphorylated tau.

Furthermore, AD has a long preclinical phase (Figure 2) consisting of three stages. In the first stage, monomeric and oligomeric Aβ aggregates inside neurons and subsequently onto neuronal surfaces and synapses as the concentration in the CSF reservoir diminishes. At this stage, current methods cannot detect the changes caused by Aβ aggregation in neurons and synapses (Hane et al., 2017). During the second stage, certain CSF biomarkers such as increased CSF tau, hypometabolism in the posterior cingulate, and cortical thinning become detectable (Hane et al., 2017). In the third stage, the patient experiences subtle symptoms while CSF Aβ decreases and CSF tau increases (Hane et al., 2017). Therefore, biomarker trajectories may differ as a function of the stage to which patients belong along the neuropathological cascade.

### Neuroimaging Biomarkers in AD

#### Imaging Amyloid and Tau Burden

Substantial advances have been made in the detection AD biomarkers using neuroimaging. In terms of imaging amyloid burden, positron emission tomography (PET) scans with radiolabeled tracers specific to Aβ have become fairly common in AD research. PET amyloid ligands allows for quantification of amyloid deposition in patients, and binding of these ligands predates the development of clinical symptoms of AD by 7–15 years (Jack et al., 2013; Roe et al., 2013). [_11_C]Pittsburgh Compound-B (PiB), a derivative of the fluorescent benzothiazole dye thioflavin T, enables for non-invasive imaging of fibrillar Aβ deposits (Klunk et al., 2004; Johnson et al., 2009). Importantly, this imaging tool is only able to detect extracellular Aβ deposition, and not intracellular Aβ accumulation. The Alzheimer’s Disease Neuroimaging Initiative (ADNI) suggests that PiB can predict cognitive decline and brain atrophy in patients with mild cognitive impairment (MCI; represents a transition toward diagnosable dementia) (Weiner et al., 2010). Amyloid imaging is now usually performed by the use of fluorine-based tracers (_18_F or FDG) and points to the parietal cortices as the earliest sites of amyloid deposition (Figure 2; Dickerson et al., 2009). The specific brain regions (posterior cingulate, retrosplenial cortex, and precuneus) are heavily connected with the medial temporal lobes (MTLs) (Ranganath and Ritchey, 2012), which are sites of early AD-related neuropathology. Tau imaging, by the use of selective PET tracers, is able to detect tau depositions that follow Braak staging of NFT pathology (Figure 2; Schöll et al., 2015; Cho et al., 2016; Maass et al., 2017). PET ligands have also been developed that are specific for paired-helical filament tau (Leuzy et al., 2019; Scholl et al., 2019). Post-mortem studies of AD patients indicate that, unlike amyloid plaque deposition, NFT density correlates with neurodegeneration and cognitive impairment (Duyckaerts et al., 1987; Braak and Braak, 1997). However, disentangling primary age-related tauopathy (PART) and AD may be a great challenge as there is considerable overlap in the MTL.

#### FDG-PET – Cerebral Glucose Hypometabolism

Positron emission tomography imaging has been used to examine brain glucose abnormalities in aging, MCI and AD (de Leon et al., 1983). FDG can be used as a metabolic marker and reduced hippocampal metabolism has been observed in patients with MCI and AD (Mosconi et al., 2005; Figure 2). FDG-PET is a sensitive biomarker for neuronal and synaptic degeneration (Zimmer et al., 2017), and in line with this, research indicates that cerebral glucose hypometabolism is a downstream marker of neurodegeneration. Thus, this imaging method is able to detect patients at a later timepoint in the course of AD. Studies (Kuhl et al., 1982) have demonstrated that in older ages cerebral glucose metabolism decreases, and that the MTLs, the posterior cingulate cortex and the precuneus show the least age-dependent change. These regions express significant hypometabolism in AD (Márquez and Yassa, 2019), and Mosconi et al. (2008) showed that FDG-PET could be used to differentiate healthy subjects from AD patients with 98 to 99% specificity. Recent work by ADNI 2 PET Core has examined how FDG-PET and amyloid PET can be combined to track progression of AD. For instance, they demonstrated that amyloid PET is negatively associated with temporoparietal metabolism (Landau et al., 2012). Amyloid PET is associated with cognitive change in healthy subjects, whereas FDG-PET imaging is able to demonstrate cognitive change in MCI patients (Jagust et al., 2015). This is consistent with the spatiotemporal progression model of AD (Figure 2), where amyloid progression precedes neurodegeneration.

#### Imaging Connectivity – Resting-State Functional Magnetic Resonance Imaging

Functional magnetic resonance imaging (fMRI) techniques use blood-oxygenation-level-dependent (BOLD) contrast, which is associated with neuronal population activity. Resting-state fMRI studies examine the correlation of the BOLD signal and anatomical regions of interest at a temporal scale by analyzing spontaneous fluctuations in brain connectivity (Biswal et al., 1995; Fox and Raichle, 2007). In preclinical stages of AD, resting-state fMRI signals have been linked to metabolic changes (indexed by PET) and found to precede neurodegeneration (Sheline and Raichle, 2013). Therefore, this imaging tool is able to detect patients at some point immediately before or after amyloid plaque deposition, prior to the development of NFTs and associated neurodegeneration. Most of these analyses have focused on the default mode network (Gusnard et al., 2001; Raichle et al., 2001), a network consisting of the MTL, the medial prefrontal cortex, posterior cingulate cortex, anterior cingulate cortex, parietal cortex, and precuneus (Greicius and Menon, 2004; Buckner et al., 2008). These regions overlap with the spatial pattern of amyloid and tau pathology (Buckner et al., 2008). In addition to changes in the default mode network, some studies have suggested that connectivity within the MTL may also be disrupted in AD (Yassa et al., 2011), such as the connectivity between EC and hippocampus [dentate gyrus (DG) and cornu ammonis field 3 (CA3)].

#### Cortical Thinning and Volume Loss – Structural MRI

Compared with functional imaging modalities, structural MRI provides an overview of anatomical changes in high resolution. Research has shown that in AD patients there is a decrease in brain volume associated with cortical thinning and gyral loss (Figure 2; Uylings and de Brabander, 2002), especially in the prefrontal cortex and hippocampus (Jack et al., 2000; Raz et al., 2005). Thus, this imaging tool can detect patients in which neurodegeneration has begun to occur. Studies in aged rodents and monkeys have demonstrated that hippocampal cells do not undergo frank cell loss with healthy aging (Rapp and Gallagher, 1996; Rasmussen et al., 1996; Rapp et al., 2002); in contrast, this is observed in the prefrontal cortex (Peters et al., 1994; Smith et al., 2004; Stranahan et al., 2012). Recently, cortical thinning of the EC has been shown to be a sensitive marker for structural alterations in both patients with MCI and AD (Holland et al., 2012a). EC thickness has been found to diminish prior to, and thereby predict, hippocampal atrophy (Desikan et al., 2010, 2011, 2012; Eskildsen et al., 2013). Several recent studies using the ADNI data have shown that older adults with CSF Aβ and phosphorylated-tau (p-tau) present with volume loss in EC (Desikan et al., 2012; Holland et al., 2012b).

#### White Matter Integrity – Diffusion Tensor Imaging

Studies using diffusion tensor imaging (DTI) in MCI and AD patients show a decrease in brain white matter integrity but with most prominent changes in MTLs (Bozzali et al., 2002; Naggara et al., 2006; Xie et al., 2006; Huang et al., 2007; Chua et al., 2008). DTI studies have focused primarily on the fornix as this region links the limbic system with the rest of the brain. Fornix lesions have been found to reproduce memory and learning deficits linked to hippocampal damage in rats (Sutherland et al., 1982; McDonald and White, 1993) and monkeys (Gaffan et al., 1984; Gaffan, 1992, 1994). The perforant path connects EC layer II neurons to the hippocampal DG and CA3 (Witter, 2007) and is critical for normal hippocampal function (Hyman et al., 1986). The integrity of this pathway is reduced in aged rats with memory loss (Geinisman et al., 1992; Smith et al., 2000). Perforant path lesions also result in EC layer II neuronal loss (Peterson et al., 1994), i.e., at the site where neurodegeneration is first observed in AD patients. Thus, similarly to structural MRI, this imaging tool can detect AD patients at the timepoint at which neurodegeneration has occurred. *In vivo* biomarkers, such as those derived from brain imaging, are crucial for accurate diagnosis of AD, but does not support diagnosis during preclinical stages. Additionally, molecular imaging is expensive and not easily accessible to the clinical population.

### CSF Biomarkers in AD

The current approach to diagnosing AD patients involves assessing patient history, clinical examinations, and detection of underlying pathology using biomarkers during stages of the disease (Ramesh et al., 2018b), with the latter having a diagnostic accuracy between 82 and 84% (Engelborghs et al., 2008). The clinical staging of AD usually lasts about 9–10 years (Heyman et al., 1996), however, researchers have found that the neuropathology of AD starts 20–30 years before the onset of clinical symptoms (Selkoe, 2001; Sperling et al., 2011). Thus, it is likely that, with current means, clinical diagnosis is only feasible at a late stage of the disease. Imaging tools are invaluable methods to diagnose AD patients, but additional methods are needed to detect AD pathology at an earlier stage of the disease cascade, where intervention may be able to delay, even, halt disease progression. By developing better screening and detection tools, early interventions at the preclinical stages of the disease should be possible.

Clearance of abnormal proteins by drainage into the CSF is an endogenous neuroprotective function of the brain. Clinical AD diagnosis is conducted by sampling CSF and analyzing aberrant protein levels within the sample. CSF fills the ventricular system in the brain and spinal cord (Barten et al., 2017) and research evidence suggests that the composition of CSF at any given time reflects true biochemical changes that occur in the brain (Lee et al., 2019). Most of the CSF is generated by the choroid plexus but a significant fraction derives from the interstitial fluid (ISF) in the brain and spinal cord parenchyma. ISF is the circulating CSF that bathes brain tissue (Barten et al., 2017), whereas the choroid plexus connects to nearby permeable capillaries with tight junctions and produces CSF using the aquaporin-1 water channel as well as directional ionic transporters (Speake et al., 2001; Brinker et al., 2014). In terms of CSF production and volume, studies have shown that it can change with age, disease, and time of day. For instance, CSF production increases from 0.4 to 1.4 μL/min between 8 and 12 weeks of age in the rat (Karimy et al., 2015). Interestingly, CSF volume has been found to increase during neurodegeneration (Barten et al., 2017), which may be related to the increase in atrophy and substance loss of the brain. It is therefore vital to keep these changes in CSF production in mind when comparing healthy subjects to AD patients, and when comparing preclinical with clinical findings.

### Temporal Course of AD Biomarkers

**FIGURE 3:**
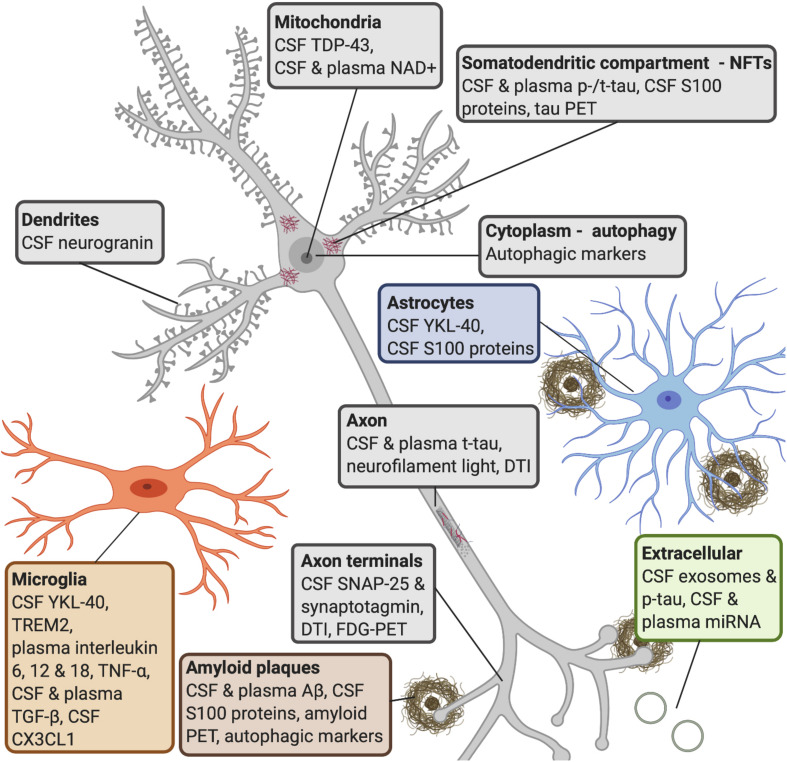
AD molecular processes that can be detected by biomarkers. *Amyloid plaques:* a widely used biomarker for diagnosis in AD is the concentration of CSF Aβ42 and Aβ40 (Rogeberg et al., 2015). Studies have shown that CSF Aβ42 can detect amyloid pathology earlier than amyloid PET imaging (Palmqvist et al., 2016). Some research shows that serum Aβ42 levels do not correlate with CSF levels (Liu et al., 2004), whereas others have found that plasma Aβ can be measured with good sensitivity (Lee et al., 2019). Several S100 proteins (S100B, S100A1, S100A6, S100A8, S100A9, and S100A12) are found within amyloid plaques and in astrocytes and/or microglia near amyloid deposits (Boom et al., 2004; Shepherd et al., 2006; Walker et al., 2006; Ha et al., 2010; Afanador et al., 2014; Lodeiro et al., 2017). *NFTs:* increased CSF tau is a sensitive biomarker for neurodegeneration, but CSF p-tau is more specific to neurodegeneration linked to AD (Lewczuk et al., 2004; Blennow et al., 2015). P-tau is secreted via exosomal release, and reaches the CSF (Saman et al., 2012). Increased levels of CSF t-tau and p-tau can predict the progression of cognitive symptoms better than CSF Aβ42 (El Kadmiri et al., 2018), but the diagnostic utility of CSF t-tau and p-tau are improved when measured in combination with Aβ42 (Dubois et al., 2014). Increased plasma tau observed in AD patients compared to MCI patients and healthy controls (Mattsson et al., 2016; Pase et al., 2019). S100B and S100A9 are found within NFTs (Sheng et al., 1994, 1997; Shepherd et al., 2006). *Autophagy:* late stages of autophagy is disrupted in AD patients, as an accumulation of autophagic vesicles can be observed in dystrophic neurites (Komatsu et al., 2006), and are observed prior to extracellular Aβ deposition (Mehrpour et al., 2010; Nixon and Yang, 2011). *Microglia:* YKL-40 is expressed by microglia. CSF TREM2 is associated with higher CSF t-tau and p-tau levels, probably reflecting a corresponding change in microglia activation in response to neurodegeneration (Suarez-Calvet et al., 2016). It has been shown that AD patients have higher levels of interleukin-6, 12, and 18, TNF-α and TGF-β, in blood, and higher levels of TGF-β in CSF, compared to healthy controls (Swardfager et al., 2010). Decreases in CSF neuronal CX3CL1 is found in AD patients (Perea et al., 2018). *Astrocytes:* YKL-40 is expressed in astrocytes near Aβ plaques (Craig-Schapiro et al., 2010) and correlates positively with tau pathology (Querol-Vilaseca et al., 2017; Janelidze et al., 2018). *Axon terminals:* CSF levels of SNAP-25 (Brinkmalm et al., 2014; Sutphen et al., 2018) and synaptotagmin (Öhrfelt et al., 2016) have been found at elevated levels in patients with AD or MCI compared with control subjects. Synaptic neurodegeneration can be detected by DTI or FDG-PET (Shen et al., 2018). *Dendrites:* increased CSF neurogranin is found in MCI and AD patients as compared with healthy controls (Thorsell et al., 2010; De Vos et al., 2015). *Axon:* increased neurofilament light is observed in response to axonal damage, which occurs in AD. The core CSF biomarkers (Aβ42, t-tau, and p-tau) and CSF neurofilament light levels strongly correlated with AD (Olsson et al., 2016). Blood levels of this protein strongly correlate with its CSF levels (Gisslen et al., 2016; Kuhle et al., 2016; Rojas et al., 2016). *Mitochondria:* studies have shown that TDP-43 contributes to neuroinflammation and may have a role in mitochondrial and neuronal dysfunction (James et al., 2016). NAD+ levels can be detected in CSF and plasma in early AD. *Extracellular:* miRNAs released from exosomes appear to be associated with neurodegenerative aspects in AD (Wang et al., 2008, 2012; Chen et al., 2017). Studies have reported that changes in levels of blood miRNA distinguished AD patients from healthy controls with 93% accuracy (Leidinger et al., 2013; Swarbrick et al., 2019). AD, Alzheimer’s disease; CSF, cerebrospinal fluid; Aβ, amyloid-β; PET, positron emission tomography; NFTs, neurofibrillary tangles; p-tau, phosphorylated tau; t-tau, total tau; MCI, mild cognitive impairment; TREM2, triggering receptor expressed on myeloid cells 2; TNF-α, tumor necrosis factor-α; TGF-β, transforming growth factor-β; CX3CL1, CX3 chemokine ligand 1; SNAP-25, synaptosomal-associated protein 25; TDP-43, transactive response element (TAR) deoxyribonucleic acid (DNA)-binding protein 43; NAD+, oxidized nicotinamide adenine dinucleotide; miRNA, microRNA.

Amyloid-β level changes is the first biomarker abnormality seen in AD patients, which can either be in the form of an upregulation in plasma and CSF in cognitively normal individuals (Figure 2). The increased levels seen in CSF Aβ40 and Aβ42 in AD patients is thought to reflect extracellular Aβ deposits prior to the accumulation of amyloid plaques (Murphy and LeVine, 2010). However, it is important to note that Aβ oligomers can form intracellularly before being deposited extracellularly, and currently this cannot be detected with existing biomarkers. Moreover, Aβ deposition detected by PET ligands can be seen as early as 15 years prior to onset of AD symptoms (Figure 2; Shen et al., 2018). The next stage of biomarker alteration include neuronal injury, shown by increased levels of CSF total tau protein (t-tau) and tau phosphorylated at threonine 181 (p-tau181/p-tau), and brain atrophy revealed by structural MRI, and synaptic loss and neurodegeneration detected by DTI or FDG-PET (Figures 2, 3; Shen et al., 2018).

### Core CSF Biomarkers for Diagnosis

Currently, CSF biomarkers are the only variety of fluid markers used for diagnosis of early AD, however, they have proven difficult to implement in the clinic due to their limited accessibility and the invasive nature of CSF collection (Lee et al., 2019). There are three core CSF biomarkers for AD diagnosis; Aβ42, t-tau, and p-tau (Shen et al., 2018). Using a combination of the core AD biomarkers is a better approach compared to using the biomarkers individually, especially for differential diagnosis (Engelborghs et al., 2008). Lower concentrations of CSF Aβ42 and higher concentrations of t-tau have been used to distinguish AD patients from healthy age-matched controls and to predict the conversion of MCI to AD (Frölich et al., 2017). To develop a non-invasive and effective measure of preclinical stages in AD, early abnormal AD biomarkers in preclinical models and patients need to be translated and assessed, followed by methodological developments of screening tools that are successful in system models that mirror the disease progression seen in patients.

## Classification of AD Biomarkers

Alzheimer’s disease biomarkers may be open to different interpretations, however, there is an international classification system proposed by the National Institute of Aging (NIH) and the Alzheimer’s Association (NIA-AA) that can aid in grouping them. In 2018, CSF biomarkers could be used in conjunction with neuroimaging for the first time to diagnose AD patients (Lee et al., 2019). The *A/T/N system* (Table 1) is a suggested grouping by Jack et al. (2016) based on the framework from the NIH and NIA-AA, where the *A* refers to the Aβ pathology measured either by PET or CSF Aβ42, the *T* represents tangle pathology and is assessed by either PET or CSF p-tau, and the *N* stands for neurodegeneration or neuronal injury detected by either FDG-PET, structural MRI, or CSF t-tau (Jack et al., 2016). Imaging techniques have found amyloid PET to be most reliable, whereas MRI and FDG-PET scans often are unable to distinguish AD more from other neurodegenerative disorders (Johnson et al., 2012). It is important to note that fluid biomarkers are more available and affordable compared to MRI and PET (Lee et al., 2019).

**TABLE 1:** AT(N) biomarker grouping of the NIA-AA framework.

**Biomarker class**	**CSF marker**	**Imaging marker**
Amyloid (A)	CSF Aβ42 or Aβ42:Aβ40 ratio	Amyloid PET
Tau (T)	CSF p-tau	Tau PET
Neurodegeneration (N)	CSF t-tau	Anatomic MRI; FDG-PET

*Adapted from Jack et al. (2016). NIH, National Institute on Aging; NIA-AA, Alzheimer’s Association; CSF, cerebrospinal fluid; Aβ, amyloid-β; p-tau, phosphorylated tau; t-tau, total tau; PET, positron emission tomography; MRI, magnetic resonance imaging; FDG, fluorine-based tracers.*

### Aβ as a Biomarker for AD

Amyloid-β production occurs at the C-terminal fragment of amyloid precursor protein (APP) by cleavage of APP by β-secretase (BACE1) to form C99, followed by cleavage of C99 by presenilin (PSEN) 1 or PSEN2, two enzymatic components of γ-secretase (Haass and De Strooper, 1999). Following production, Aβ42 aggregates and accumulates intracellularly and/or extracellularly until a critical threshold is reached, where CSF Aβ42 decreases as the peptide sequesters in amyloid plaques in the brain parenchyma (Cirrito et al., 2003; Hong et al., 2011). The long delay in the emergence of plaque deposits even in the presence of increased Aβ42, suggests an initial slow process where monomeric Aβ forms small aggregates, followed by further Aβ polymerization (Harper and Lansbury, 1997). The AD field cannot yet explain exactly how Aβ pathology initiates, but further research on the generation of intracellular Aβ monomers, their recycling, and their aggregation into oligomeric Aβ may yield some answers.

One of the most widely used biomarkers in AD diagnostic research is the measurement of Aβ42 and Aβ40 in CSF (Figure 3; Rogeberg et al., 2015). Although Aβ40 is present at about 10–20 times higher concentration in CSF, Aβ42 is more prone to aggregate and shown to correlate better with AD neuropathology (Mehta et al., 2001; Hellstrand et al., 2010; Murphy and LeVine, 2010; Savage et al., 2014). It has been found that cognitively normal older adults that developed dementia in older ages had low CSF Aβ42 but not Aβ40 levels (Blennow et al., 2015). These findings can be explained in part by how Aβ aggregates to form soluble oligomers, which can exist in multiple forms and are neurotoxic (Haass and Selkoe, 2007), and which finally conform to diffuse and dense plaques. Recent studies support the notion that accumulation of the Aβ peptide arises from an imbalance in the production and clearance of Aβ and that the ability to clear Aβ diminishes with age (Wildsmith et al., 2013). An attractive early biomarker for AD is CSF Aβ42, given that both CSF t-tau and p-tau changes occur at a later time point in the disease process closer to clinically detectable dementia (Buchhave et al., 2012). Furthermore, measurement of the CSF Aβ42:Aβ40 ratio is superior to Aβ42 alone when distinguishing between MCI patients who progress and those that do not progress to AD dementia (Hansson et al., 2007; Lee et al., 2019). When comparing Aβ fluid biomarkers and imaging biomarkers, studies have shown that CSF Aβ42 can detect amyloid pathology earlier than amyloid PET imaging (Figure 3; Palmqvist et al., 2016).

There appears to be a lack of consensus regarding CSF Aβ concentrations in AD patients. For instance, researchers have found that CSF Aβ42 concentrations increase (Nakamura et al., 1994; Bouwman et al., 2007), decrease (Kanai et al., 1998; Tapiola et al., 2000; Wahlund and Blennow, 2003; Mollenhauer et al., 2005; de Leon et al., 2006; Beckett et al., 2010), or experience no significant change (Andreasen et al., 1998, 1999a; Hoglund et al., 2005; Andersson et al., 2008; Brys et al., 2009; Stomrud et al., 2010) during the disease. The prevailing explanation for a reduced amount of Aβ in later stages of AD is that as the pathology progresses, more Aβ, especially Aβ42, aggregates into plaques in the brain, which effectively means that less Aβ can diffuse from the brain to the CSF. Another explanation may be that Aβ first accumulates intracellularly, and neurodegeneration releases Aβ in the extracellular compartment, increasing CSF Aβ levels, and subsequently accumulates onto neuronal surfaces and in synapses as it clears away from the CSF. As neurodegeneration occurs, less Aβ is produced and therefore smaller amounts will accumulate in the CSF and subsequently in the brain, where Aβ will reach plateau levels. Alternatively, reduced CSF Aβ42 might follow neuronal dysfunction, which results in decreased metabolism of APP and Aβ. It is important to note that this is unlikely in transgenic mice, as Aβ42 levels in the CSF decline, while its levels in the brain keep rising. Also, when above a certain level, parts of CSF Aβ42 may aggregate into a large assembly that antibodies of currently used enzyme-linked immunosorbent assay (ELISA) kits cannot capture (Pitschke et al., 1998; Liu et al., 2004). These seemingly disparate findings regarding CSF Aβ concentrations may therefore reflect different timepoints of the disease progression.

With regard to studies using Aβ as a fluid biomarker for AD, studies have shown that concentrations of CSF Aβ42 increased between 5 and 7 months of age, but not between 8 and 13 months of age in an APP/PS1 mouse model (Liu et al., 2004). This was despite a rapid increase in brain levels of Aβ42 (Liu et al., 2004). However, between 6 and 9 months of age in another APP/PS1 mouse model with a more aggressive AD phenotype a decline in CSF Aβ42 levels was reported (Liu et al., 2004). Based on these findings, it appears that CSF Aβ42 may initially reflect the rate of Aβ42 production (most likely in the synaptic cleft), but after reaching a critical threshold, CSF Aβ42 levels stay in equilibrium until plaque formation leads to their decrease (Liu et al., 2004). Consistent with this notion, CSF Aβ42 levels have a strong link with the deposition of amyloid plaques, whereby an inverse correlation between Aβ42 levels, plaques (Strozyk et al., 2003) and amyloid PET is observed (Toledo et al., 2015; Leuzy et al., 2016; Niemantsverdriet et al., 2017). This implies that as Aβ aggregates and forms plaques in the brain, lower levels of the protein diffuse into the CSF (Lee et al., 2019).

Compared to tau as a fluid biomarker, the drop in CSF Aβ42 should precede the increase in CSF tau proteins. This notion is supported by biomarker studies in sporadic AD patients demonstrating that a decrease in CSF Aβ42 was the earliest change reported (Skoog et al., 2003; Gustafson et al., 2007), while in patients with familial AD reductions in CSF Aβ42 and elevations in tau occur around 10–15 years prior to symptom development (Bateman et al., 2012; Ringman et al., 2012). Similar to Aβ, tau can also change its conformation to prion-like oligomers and there is evidence for misfolded Aβ initiating tau misfolding (Pulawski et al., 2012; Nussbaum et al., 2013). Therefore, Aβ appears to be an initiator of tau pathology and subsequent neurodegeneration.

### Tau as a Biomarker for AD

Tau is a microtubule-associated protein comprising six human isoforms and is located in neuronal axons (Barten et al., 2011; Khan and Bloom, 2016). A characteristic of many neurons in AD is that tau is hyperphosphorylated and translocated from axons to the somatodendritic compartment, where it becomes misfolded and aggregates. Tau aggregates develop intracellularly and may thus trap functional proteins adding to microtubule destabilization, cellular dysfunction and eventually neurodegeneration (Benilova et al., 2012). Intracellular trafficking is dependent on the phosphorylation of tau in order to separate tau from microtubules, allowing transport, followed by dephosphorylation in order to return tau into microtubules (Avila et al., 2004). It has been proposed that Aβ pathology drives the abnormal phosphorylation of tau in AD (Bakota and Brandt, 2016; Khan and Bloom, 2016). However, transgenic mice modeling Aβ pathology alone do not develop NFTs endogenously (Lee and Trojanowski, 2001), while intracerebral injections of human mutated and/or aggregated tau are necessary to observe this neuropathological hallmark (Clavaguera et al., 2013; Ahmed et al., 2014; Kaufman et al., 2017; Lewczuk et al., 2017; Mudher et al., 2017; Narasimhan et al., 2017; He et al., 2018). Tau aggregates and NFTs are produced in the cytoplasm under pathological conditions, when tau changes its conformation from a highly soluble state to one with a high β-sheet content and is hyperphosphorylated (Yamada et al., 2015). Studies have shown that the elevation in CSF tau in AD is due to axonal loss and neuronal death, leading to the release of the intracellular protein (Blennow and Hampel, 2003; Hampel et al., 2010). However, despite significant neurodegeneration and tau pathology, CSF tau is not elevated in other pure tauopathies (Grossman et al., 2005; Bian et al., 2008). This suggests that cell death may not be the only mechanism responsible for CSF tau elevations in AD (Jack et al., 2010).

When using tau as a fluid biomarker for AD, increased CSF concentrations of the protein constitutes a sensitive marker for neurodegeneration, but an entirely unspecific one for AD (Figure 3). However, the increased concentration of p-tau molecules seems much more AD specific (Figure 3; Lewczuk et al., 2004; Blennow et al., 2015). P-tau is secreted via exosomal release, and reaches the CSF (Saman et al., 2012; Figure 3). CSF p-tau levels are usually stable in other dementias, whereas both CSF p-tau and t-tau levels can be used to distinguish AD patients from healthy controls, suggesting that CSF tau is an important biomarker for differential dementia diagnosis (Blennow et al., 2015). In addition, high CSF t-tau and p-tau can predict the progression of cognitive symptoms better than CSF Aβ42 (El Kadmiri et al., 2018; Figure 3). Researchers have found correlations between p-tau in CSF and NFTs in the brain (Clark et al., 2003; Buerger et al., 2006; Tapiola et al., 2009; de Souza et al., 2012), and CSF t-tau has been found to correlate with neurodegeneration (Lee et al., 2019). Importantly, the utility of CSF p-tau and t-tau for AD diagnosis is markedly improved when measured together with CSF Aβ42 (Figure 3; Dubois et al., 2014).

To examine CSF tau levels in rodents, researchers have used P301S human tau transgenic mice and found that ISF tau was at fivefold higher levels compared to endogenous tau, in line with its elevated levels of expression (Yamada et al., 2011). It is important to keep this in mind when comparing CSF tau between rodents and patients, as the CSF tau from rodents might be contaminated by ISF tau levels, and the CSF levels may not reflect the actual tau levels expressed in the brain. Studies have found that tau in brain tissue is approximately 50,000-fold more abundant than its levels in the CSF (Barten et al., 2011). In humans, patients with sporadic AD display longitudinal increases in tau when low levels of tau were detectable early in the disease course, but no differences (or increases) have been observed in tau in patients with high levels of tau at baseline (Kanai et al., 1998; Sunderland et al., 1999).

Compared to Aβ, which aggregates broadly in the brain parenchyma and the perivascular space, tau readily drains into CSF. In contrast to Aβ, tau levels increase in the CSF with the progression of AD (Olsson et al., 2016). Changes in CSF Aβ42 precede changes in CSF tau, consistent with the proposition that Aβ affects and drives CSF tau levels (Jack et al., 2010). However, further research is needed to explain whether Aβ and tau pathology represent early initiators of neurodegeneration and cognitive decline, or merely downstream effects of other early pathophysiological events in AD.

### Neuroinflammatory Biomarkers

Inflammation is now considered another core feature of AD as it relates to the pathogenesis of the disease and also serves as a link between amyloid plaques and NFTs (Akama and Van Eldik, 2000; Akiyama et al., 2000). Inflammatory response has now been reported in post-mortem tissues of AD patients (Gomez-Nicola and Boche, 2015) and is routinely observed in preclinical models. The presence of inflammation in the brain of AD patients was initially thought to be a consequence of the accumulating neurodegeneration present at late stages of the disease. However, a substantial body of research now demonstrates that a persistent immune response in the brain not only is associated with neurodegeneration, but it also exacerbates Aβ and tau pathology (Figure 1). Inflammatory markers may be able to give evidence to intracellular abnormalities early in the course of AD, prior to extracellular Aβ deposition (marked by an increase in CSF Aβ40/Aβ42 and amyloid PET) as evidence points to a dysfunction in autophagic processes in AD patients (Figure 1; Menzies et al., 2015). Moreover, there have been reports of immune-related proteins and cells opposed to amyloid plaques (Figure 1; Griffin et al., 1989). In line with this, it has been suggested that inflammation may provide a link between the initial Aβ pathology and subsequent development of NFTs (Kitazawa et al., 2004; Rhein et al., 2009; Garwood et al., 2011; Nisbet et al., 2015).

#### Autophagic Markers

One way for intracellular accumulation of Aβ peptides to occur is through a disruption in the autophagic and lysosomal clearance systems. In this way, autophagic markers could ultimately serve as the earliest biomarker to diagnose AD patients, as this process may likely initiate the aggregation of monomeric Aβ into oligomeric Aβ intracellularly (Figure 1). Autophagy is a complex process, in which a vesicle known as the phagophore elongates around the cytoplasmic components selected for degradation. The recognition of these components are dependent on the lipidated form of the microtubule-associated protein light chain 3 (LC3) (Milisav et al., 2015). The late stage of autophagy depends on the successful fusion of the autophagosome with the lysosome, which then degrades and recycles the autophagosome cargo. There is evidence supporting that the late stage of autophagy is disrupted in AD, as accumulation of autophagic vesicles can be observed in dystrophic neurites (components of dense plaques) (Komatsu et al., 2006), and these are observed prior to extracellular Aβ deposition in model systems and patients (Figure 3; Mehrpour et al., 2010; Nixon and Yang, 2011).

The above findings suggest that autophagy dysfunction leads to the accumulation of intracellular Aβ by avoiding proper degradation and/or recycling. In line with this, researchers have found that LC3-associated endocytosis is used to clear and recycle Aβ surface receptors (Heckmann et al., 2019). In model systems with LC3-associated endocytosis disrupted, an increase in extracellular Aβ deposition, NFTs, neurodegeneration and behavioral deficits was observed. Another line of research found that autophagic markers were significantly increased in AD patients compared with control subjects (Cho et al., 2019). Furthermore, other studies suggest that metabolism of Aβ and tau is crucially influenced by autophagy (Uddin et al., 2018). Recent evidence suggests that Aβ monomers and oligomers modulate autophagy differently in neurons. Monomers have been found to stimulate autophagy, increasing autophagosome rates and elevation of LC3 protein levels, while simultaneously impairing the lysosomal pathway affecting the autophagy efflux, leading to autophagosome accumulation (Menzies et al., 2015). By contrast, Aβ oligomers do not cause a significant increase in LC3 protein levels nor affect efflux of autophagic vacuoles (Menzies et al., 2015), which suggests that an increase in intracellular Aβ monomers may be the result of a defective autophagic system. This fits the proposition that autophagic disruption and accumulation of Aβ monomers constitute some of the earliest events in the AD cascade (Figure 1). The exact cascade by which autophagy can degrade amyloid plaques is still not known, however, microglial autophagy appears to play and important role.

#### Glial Cells and Markers

Neuroinflammation involving astrocytes, microglia, and secreted compounds like reactive oxygen species, cytokines, and chemokines are key pathophysiological processes assessed when diagnosing AD (Ramesh et al., 2018a). Microglia and astrocytes are the two types of glial cells primarily affected (McGeer et al., 1987; Rogers et al., 1988; Bronzuoli et al., 2016), which in turn affect the clearance and production of Aβ42 (Hickman et al., 2008; Liu et al., 2017). Glial cells also affect the development and propagation of tau pathology (Asai et al., 2015) and thus influence disease progression and severity (Block et al., 2007; Calsolaro and Edison, 2016). Importantly, if glial cells are activated for too long, they can become pro-inflammatory (Bronzuoli et al., 2016). Chitinase-3-like protein 1 (or YKL-40) is an inflammatory marker expressed by microglia and astrocytes (Figure 3). In AD, YKL-40 is expressed in astrocytes near Aβ plaques (Craig-Schapiro et al., 2010) and correlates positively with tau pathology (Figure 3; Querol-Vilaseca et al., 2017; Janelidze et al., 2018).

Another glial marker is triggering receptor expressed on myeloid cells 2 (TREM2), which is an inflammatory cell-surface receptor. Loss-of-function mutations of TREM2 are associated with an increased risk of developing AD (Suarez-Calvet et al., 2016). Researchers have found that TREM2 phagocytose Aβ in early AD stages (Jay et al., 2017). A rare mutation in the TREM2 gene affects the phagocytic activity of microglia and consequently contributes to accumulation of Aβ (Kleinberger et al., 2014; Ramesh et al., 2018b). With regard to TREM2 measured in CSF, studies have shown increased levels to be associated with higher CSF t-tau and p-tau levels, probably reflecting a corresponding change in microglia activation in response to neurodegeneration (Figure 3; Suarez-Calvet et al., 2016). Relevant research findings imply that neuroinflammation is a robust biomarker even at pre-symptomatic stages (Janelidze et al., 2018). In line with this, high levels of inflammatory biomarkers are associated with increased CSF levels of t-tau (Janelidze et al., 2018). Interestingly, PET studies have revealed increased microglial activation in the precuneus (Hamelin et al., 2016; Fan et al., 2017), a region in the default mode network that displays early Aβ deposits in AD (Palmqvist et al., 2017).

#### Cytokines

With regard to cytokines, it has been shown that AD patients have higher levels of interleukin-6, 12, and 18, tumor necrosis factor-α (TNF-α), and transforming growth factor-β (TGF-β), in blood, and higher levels of TGF-β in CSF, compared to healthy controls (Figure 3; Swardfager et al., 2010). Endothelial growth factor receptor 1 (also known as Flt-1), has been found to be upregulated in entorhinal cortical sections from human AD brains and in human microglia following treatment of Aβ42 (Ryu et al., 2009). Following neuroinflammation, oxidative stress in the intracellular environment occurs. Oxidative stress plays an important role in the early stages of AD (Oh et al., 2010) and its associated signaling cascades are being investigated and explored for biomarkers. For instance, higher reactive oxygen species levels lead to post-translational modification of proteins, toxic cell damage, fragmentation and aggregation of Aβ (Brinkmalm et al., 2014). One type of reactive oxygen species are sulfatides, which have been found to be depleted in both gray and white matter of AD patients, and results in decreased hippocampal volume and cognitive decline (Ramesh et al., 2018b).

Since neuroinflammation occurs in AD brains, the levels of several S100 proteins are increased and some of the proteins play roles related to the processing of APP, regulation of Aβ levels and tau phosphorylation. S100A1, S100A6, and S100B have been found to be involved in the disassembly of microtubules and tau protein release (Zimmer et al., 2005; Roltsch et al., 2010; Wruck et al., 2016; Sidoryk-Wegrzynowicz et al., 2017), while S100B and S100A9 are found within NFTs (Figure 3; Sheng et al., 1994, 1997; Shepherd et al., 2006). Traumatic brain injury (TBI) has been found to predispose people to developing AD (Johnson et al., 2010). Interestingly, TBI results in an increase in S100A1 and S100B levels in plasma and CSF in patients (de Boussard et al., 2005). Research has shown that levels of S100B originating from necrotic tissue might enhance or amplify neurodegeneration by apoptosis (Sedaghat and Notopoulos, 2008). Thus, various S100 proteins could be a link between neurodegenerative diseases induced by brain damage. Comorbidities often accompany a diagnosis of AD, thus the spectrum of pathological processes that can end in AD at different degrees of severity and symptomology needs to be kept in mind in order to accurately diagnose and treat patients. Additionally, S100 proteins may serve as an early biomarker for a later AD diagnosis in patients with TBI or other comorbidities that increase S100 levels.

Moreover, several S100 proteins are implicated in the amyloidogenic pathway of APP cleavage. S100A9 regulates γ-secretase and BACE1 expression and activity (Kummer et al., 2012; Li et al., 2014), and S100B and S100A1 regulate APP levels (Zimmer et al., 2005; Anderson et al., 2009; Mori et al., 2010). S100A7, S100A8, S100A9, and S100B have been found to influence Aβ levels (Qin et al., 2009; Lee et al., 2013; Lodeiro et al., 2017; Cristovao et al., 2018). Moreover, S100B and S100A6 have been found to reduce zinc levels and senile plaque load in preclinical models (Roltsch et al., 2010; Hagmeyer et al., 2017; Tian et al., 2019). S100A1, S100A9, and S100B proteins can interact and alter the aggregated Aβ and is found to co-aggregate with Aβ peptides (Zimmer et al., 2005; Shepherd et al., 2006; Ha et al., 2010; Mori et al., 2010; Chang et al., 2012; Afanador et al., 2014; Cristovao et al., 2018). In line with this, several S100 proteins (S100B, S100A1, S100A6, S100A8, S100A9, and S100A12) are present in amyloid plaques and in astrocytes and/or microglia near amyloid deposits (Figure 3; Boom et al., 2004; Shepherd et al., 2006; Walker et al., 2006; Ha et al., 2010; Afanador et al., 2014; Lodeiro et al., 2017).

#### Chemokines

In order to maintain brain homeostasis, microglia establish continuous communication with neurons and astrocytes, through the expression and secretion of chemokines (Mennicken et al., 1999). The CX3 chemokine ligand 1 (CX3CL1; or fractalkine), is predominantly expressed in neurons (Bazan et al., 1997) and interacts with the CX3 chemokine receptor 1 (CX3CR1) exclusively present in microglia (Imai et al., 1997; Maciejewski-Lenoir et al., 1999). The CX3CL1/CX3CR1 tandem allows for direct communication between neurons and microglia (Harrison et al., 1998; Sheridan and Murphy, 2013), and it has been suggested that this axis becomes impaired in AD patients (Bolos et al., 2017). Consistent with this, neuronal CX3CL1 is found to be decreased in CSF from AD patients compared to MCI and control subjects (Figure 3; Perea et al., 2018). Furthermore, researchers have aimed to regulate neuroinflammation in tau depositing mouse lines by overexpressing CX3CL1 and found that it significantly reduced tau pathology, ameliorated neuronal loss, reduced microgliosis (Nash et al., 2013) as well as rescuing cognitive function (Finneran et al., 2019). In another line of research, it has been found that when microglia are transferred from tau depositing knock-out *Cx3cr1* mice, hyperphosphorylation of endogenous murine tau is observed (Maphis et al., 2015). Disruption of CX3CL1 signaling in amyloid depositing mouse lines has shown reduced pathology due to increased microglial phagocytosis of amyloid plaques (Lee et al., 2010).

### Synaptic Neurodegeneration Markers

There is of yet no established or reliable biomarker test for synaptic degeneration, which is considered a crucial feature for the development of AD-related cognitive decline. The ability to monitor neurodegeneration as a downstream effect of synaptic dysfunction would be an important advantage for early AD diagnosis and in clinical trials related to drug testing. Synaptotagmin, a pre-synaptic calcium sensor vesicle protein, facilitates neurotransmitter release from the synaptic vesicle by exocytosis and also functions as an essential vesicle cargo molecule in hippocampal neurons (Leinenbach et al., 2014). Various studies have shown a decrease in synaptotagmin-1 in AD patients (Mattsson et al., 2011).

Another marker for synaptic degeneration is synaptosomal-associated protein 25 (SNAP-25), which is an essential component of the soluble N-ethylmaleimide-sensitive fusion protein attachment protein receptors (SNARE) complex that mediates synaptic communication by initiating fusion of synaptic vesicles (Olsson et al., 2016). A negative correlation has been found between SNAP-25 and cognitive decline, suggesting that this is a promising novel CSF biomarker for AD (Andreasen et al., 1999b). Overall, CSF levels of SNAP-25 (Brinkmalm et al., 2014; Sutphen et al., 2018) and synaptotagmin (Öhrfelt et al., 2016) have been assessed and found at elevated levels in patients with AD or MCI compared with control subjects (Figure 3). Another marker for synaptic neurodegeneration is neurogranin, which plays an important role in synaptic plasticity and long-term potentiation processes (Geppert et al., 1994; Sudhof and Rizo, 1996; Thorsell et al., 2010). Neurogranin has also been found at increased levels in CSF of patients with MCI and dementia due to developing AD as compared with healthy controls (Figure 3; Thorsell et al., 2010; De Vos et al., 2015). CSF neurogranin concentrations have been found at increased levels in patients that have reached the threshold for Aβ PET detection (Palmqvist et al., 2019), and also CSF neurogranin and tau levels have been found to correlate strongly (Thorsell et al., 2010; De Vos et al., 2015).

### Novel Biomarkers for AD

Many biomarkers are now being investigated as complimentary to the core AD biomarkers. One such biomarker is neurofilament light, which is a marker of neuronal integrity reflecting axonal damage of the subcortical white matter (Petzold, 2005; Jahn and Fasshauer, 2012; Neselius et al., 2012; Kuhle et al., 2015; Zetterberg et al., 2016). Neurofilament light is released from axons into the extracellular space during healthy aging, which results in increased CSF level concentrations (Figure 3). The release of neurofilament light is accelerated during axonal damage, which occurs in AD (Figure 3). A recent meta-analysis showed that the core CSF biomarkers (Aβ42, t-tau, and p-tau) and CSF neurofilament light levels strongly correlated with AD (Figure 3; Olsson et al., 2016). Importantly, CSF neurofilament light levels have been shown to be higher in AD (Sjogren et al., 2001; Pijnenburg et al., 2007; Zetterberg et al., 2016; Alcolea et al., 2017; Lista et al., 2017). Blood neurofilament levels strongly correlate with CSF levels (Figure 3; Gisslen et al., 2016; Kuhle et al., 2016; Rojas et al., 2016), and blood neurofilament light concentrations have been found at increased levels in many forms of neurodegenerative disease (Gisslen et al., 2016; Kuhle et al., 2016; Rohrer et al., 2016; Rojas et al., 2016; Steinacker et al., 2016, 2017; Weydt et al., 2016; Mattsson et al., 2017a) and have proven almost as reliant as CSF analysis in monitoring treatment outcome in patients (Bacioglu et al., 2016; Disanto et al., 2017). Another novel biomarker is the transactive response element (TAR) deoxyribonucleic acid (DNA)-binding protein 43 (TDP-43) protein, which can become pathologic if triggered by Aβ peptides. Studies have shown that TDP-43 contributes to neuroinflammation and may have a role in mitochondrial and neuronal dysfunction (Figure 3; James et al., 2016). In accordance with this, TDP-43 pathology has been observed in some AD cases (Amador-Ortiz et al., 2007; Chang et al., 2015; James et al., 2016). Another novel biomarker is oxidized nicotinamide adenine dinucleotide (NAD+; involved in mitochondrial homeostasis), which has been found to decrease during healthy aging, but even more rapidly in neurodegenerative diseases (Figure 3; Lautrup et al., 2019). This could be related to the observed decrease in neuronal metabolism that occurs during healthy aging, but this is accelerated in the AD brain. NAD+ levels can be detected in CSF and plasma early during the disease progression, and in combination with core biomarkers, it presents as a novel preclinical biomarker for AD (Figure 3).

Neuropathology is associated with a distinct subset of cells in specific regions in the brain and this makes the identification of relevant biomarker molecules a challenge. The transport of macromolecules from the brain to the CSF and blood, mediated by extracellular vesicles, presents a promising source of central nervous system (CNS)-specific biomarkers (Thompson et al., 2016). One such trafficking macromolecule is exosomes, which can be picked up in CSF. An increasing body of evidence suggests that exosomal proteins and microRNAs (miRNAs) may constitute novel biomarkers for clinical AD diagnosis (Van Giau and An, 2016). miRNAs released from exosomes appear to be associated with neurodegenerative aspects in AD (Figure 3; Wang et al., 2008, 2012; Chen et al., 2017). miRNAs are a class of small non-coding RNAs which regulate over 50% of protein-coding genes, and miRNA-107 has been found to be downregulated in AD brains (Van Giau and An, 2016; Fransquet and Ryan, 2018; Ramesh et al., 2018b). Accumulating evidence presents that miRNAs regulate Aβ production, NFT formation, and neurodegeneration by targeting different genes (Wang et al., 2008; Van Giau and An, 2016). Research has also identified BACE1 as a target of miRNA-107, connecting the level of miRNA-107 to Aβ formation and neuronal pathogenesis (Wang et al., 2008).

## CSF and Blood-Based Biomarkers

Although protein content is lower in CSF compared to blood, CSF holds great value for developing consistent biomarkers for AD as it reflects biochemical changes in the brain by direct interaction with the extracellular space (Hampel et al., 2012). At present, there is no approved blood biomarker for AD (Stadtman and Levine, 2003). It is important to note, however, that blood biomarkers have lower sensitivity and specificity than CSF biomarkers, and this can be attributed to the fact that the blood-brain barrier (BBB) prevents diffusion of analytes into the blood via a filtering mechanism (Lee et al., 2019). Furthermore, one complication of measuring CNS biomarkers in blood is that many of the analytes are produced in the periphery as well as in the brain, and thus the source of detectable change may be difficult to determine (Barten et al., 2017). However, there is currently an urgent need within the field to develop blood-based biomarkers which are inexpensive and which can detect early neuropathological changes in AD (Hane et al., 2017). For instance, detection of autophagic markers in plasma could serve as an early biomarker for AD (Cho et al., 2019). Blood test sampling is routinely performed in the clinic, it is minimally invasive, cheap and suitable for recurrent measurements (Shen et al., 2018).

When comparing Aβ levels in blood and CSF, some researchers have found that serum Aβ42 levels do not correlate with CSF levels (Figure 3; Liu et al., 2004). However, contrary to this, others have found that plasma Aβ can be measured with good sensitivity (Figure 3; Lee et al., 2019). Aβ can easily penetrate the BBB and is therefore an attractive blood biomarker candidate (Lee et al., 2019). Indeed, studies have shown that cerebral amyloid deposits may be sourced in the periphery, while other studies suggest that amyloid deposits in cerebral vessels may originate from circulating Aβ peptides (Yankner and Mesulam, 1991; Chen et al., 1995; DeMattos et al., 2002; Lee et al., 2019). Plasma Aβ and Aβ-approximate peptide concentrations have been reported to be consistent with amyloid PET results (Kaneko et al., 2014). Moreover, levels of Aβ42 and the ratio of Aβ42:Aβ40 in plasma have been shown to correlate with CSF concentrations and with amyloid PET (Janelidze et al., 2016; Verberk et al., 2018). However, it is important to note that some researchers have found that reduction of Aβ in the periphery does not reduce brain Aβ levels (Georgievska et al., 2015) [but see Jin et al. (2017)].

To date, neurofilament light is the only biomarker that is translatable from plasma to CSF, and therefore holds great promise as a clinical tool to predict cognitive decline and neurodegeneration in AD (Figure 3; Zetterberg et al., 2016; Mattsson et al., 2017a; Lewczuk et al., 2018). Furthermore, it has been shown that measurements in blood and CSF levels strongly correlate and that neurofilament light increases coincided with the onset and progression of corresponding amyloid pathology in the brain (Figure 3; Bacioglu et al., 2016). Studies have shown that plasma neurofilament light can be used as a non-invasive biomarker that strongly correlates with neurodegeneration in human AD patients (Mattsson et al., 2019). Moreover, plasma t-tau levels can be used for screening and prognosis of cognitive decline in patients where CNS injury has been ruled out (Molinuevo et al., 2018). However, there are decreased amounts of tau in plasma compared to CSF (Barten et al., 2017; Mattsson et al., 2017b), but increases have been found in plasma of AD patients when compared to MCI patients and healthy controls (Figure 3; Mattsson et al., 2016; Pase et al., 2019). Studies looking at circulating RNA biomarkers for AD have reported that changes in levels of blood miRNA distinguished AD patients from healthy controls with 93% accuracy (Figure 3; Leidinger et al., 2013; Swarbrick et al., 2019). Therefore, blood miRNAs could be an addition to the biomarker toolbox for diagnosing AD patients. For a more extensive review on comparisons between CSF and blood biomarkers in AD, and developments in biochemical analyses of blood, see Ashton et al. (2020).

## Methods for CSF Sampling

### CSF Collection in Human Patients

The most commonly used method for sampling CSF in human patients is by lumbar puncture. Clinically, lumbar punctures are routinely performed for diagnosing multiple brain disorders (e.g., meningitis, encephalitis, multiple sclerosis) and for the administration of spinal anesthesia and chemotherapy. However, there are several limitations associated with the use of lumbar punctures, such as associated pain during and after the sampling (including post-puncture headache) in patients (Blennow et al., 2015). Additionally, CSF sampling in patients who cannot cognitively consent to the procedure is ethically problematic. One also needs to keep in mind that Aβ is higher in lumbar CSF (Brandner et al., 2014), and tau is higher in ventricular CSF (Tarnaris et al., 2011; Pyykkö et al., 2014; Herukka et al., 2015) when using this method for CSF collections. However, the timing of intraventricular CSF sampling will likely affect concentrations of Aβ and tau, for example whether the sample is taken immediately after the insertion of a ventricular catheter. Research suggests that increased ventricular CSF tau concentrations may be caused by the sampling procedure itself, whereby neurons affected by the insertion of the needle for spinal tap increasingly release tau molecules (Brandner et al., 2014). In line with this, CSF samples taken shortly after surgery often have elevated tau and neurofilament light levels (Barten et al., 2017). CSF flow rate is slower in lumbar regions compared to cephalic regions (Sweetman and Linninger, 2011), and this may additionally cause the differences in concentrations of analytes. However, contradictory evidence suggests that p- and t-tau concentrations are 20–30% lover in intraventricular CSF, compared to lumbar CSF, and that this initial upregulation post-surgery is stable in patients irrespective of brain Aβ pathology (Leinonen et al., 2019).

### CSF Collection in Animal Models

The most commonly used method for sampling CSF in rodents is collections from the cisterna magna, however, this sampling method usually constitutes a terminal procedure. Collections from the cisterna magna in preclinical models for *in vivo* sampling of CSF have proven a valuable technique for studying treatment outcomes after drug delivery to the CNS. This CSF sampling method offers the advantage of serial sampling without the cofound of anesthesia, with the added benefit of using animals as their intrinsic controls (Amen et al., 2017). Another technique involves inverting animals during CSF collection in order to drain spinal CSF into the cisterna magna (DeMattos et al., 2002). An alternative technique involves collecting CSF by puncturing the membrane by suction using a pipette (DeMattos et al., 2002; Barten et al., 2011). One major limitation of collections from the cisterna magna in preclinical models is the small volume of CSF that can be obtained. In transgenic mice, the average volume is approximately 5–15 μl for terminal sampling (Liu and Duff, 2008). For serial sampling, a maximum of 7–8 μl can be safely taken each time at an interval of 2–3 months (Liu and Duff, 2008).

Microdialysis is an alternative CSF sampling technique, which allows continuous *in vivo* sampling of molecules within the extracellular space (Takeda et al., 2011), which may help circumvent some of the above limitations. Sampling using this method relies on diffusion of analytes across a semi-permeable dialysis membrane (Takeda et al., 2011). This method is advantageous over other CSF sampling techniques as it enables serial sampling that follows the dynamic temporal alterations of a target molecule without necessitating the collection of biopsy samples or sacrifice (Meyding-Lamadé et al., 1996; Trickler and Miller, 2003; Liu et al., 2004; Liu and Duff, 2008). Importantly, each preclinical model can serve as their own intrinsic control in order to reduce inter-animal variability and the number of animals used in experiments. One limitation of this method, however, is detection of large molecules due to adsorption in tubing and the dialysis membrane, as well as low concentration of analytes in the target tissue (Ao and Stenken, 2006). Furthermore, histochemical techniques have revealed that severe gliosis around implanted devices such as microdialysis cannulas takes place at about 4 days after the surgery (Hamberger et al., 1985; Benveniste and Diemer, 1987; Benveniste et al., 1987). Reports suggest that a complete recovery of physiological functions occurs at the earliest at 5–7 days after the implantation surgery (Drijfhout et al., 1995).

## CSF AD Biomarkers and Treatment

Studies suggest that the neuropathological events that occur in AD may disturb physiological functions of the BBB and thereby distribution of drugs to the brain (Pahnke et al., 2014; Vellonen et al., 2017). Drug molecules in the peripheral circulation are controlled and limited from entry into the brain by the BBB, while dysfunction of the BBB has been associated with neurodegeneration (Vellonen et al., 2017). Furthermore, AD drugs may not be transported to their site of action due to a dysfunctional BBB. This may lead to an increase of the drug in the brain leading to unwanted effects, or decreased drug circulation leading to an insufficient response (Vellonen et al., 2017). Many agents are better dosed directly into the CSF than peripherally because of limited permeability of the drug through the BBB (Barten et al., 2017). The presence of Aβ plaques, brain atrophy and dilated ventricles in the AD brain may affect the distribution of drugs in brain tissue (Vellonen et al., 2017). CSF levels of autophagic markers, Aβ and tau may help select an appropriate AD treatment for the timepoint of diagnosis. The CSF pharmacokinetics of a treatment after administration may show how well the drug entered the CNS. Therefore, levels of CSF autophagic markers or Aβ could be a pharmacodynamic marker of inhibited Aβ production (Dockens et al., 2012; Albright et al., 2013; Coric et al., 2015), and, in a longer term, CSF tau decreases could be a downstream functional marker of reduced neurodegeneration (Riekse et al., 2006).

### Assessing Autophagy in CSF Samples

Inductors of autophagy could be used in order to halt or stop the development and progression of Aβ pathology in model systems and patients with AD. Trehalose, an inductor of autophagy, was found to significantly improve memory and learning tasks in APP/PS1 mice (Rami, 2009). Importantly, Aβ deposits were found to be significantly reduced in the hippocampus of these mice (Rami, 2009). Furthermore, the induction of autophagy by rapamycin in another model system was found to improve cognitive performance through the degradation of extracellular Aβ depositions (Rami, 2009), and has been found by others to inhibit tau pathology (Caccamo et al., 2010). In AD, changes in early endocytosis and autophagy can be detected in CSF (Armstrong et al., 2014). Moreover, some work within the AD field has focused on lysosomal proteins, as they can be found in and around amyloid plaques and is present in CSF (Cataldo and Nixon, 1990; Schwagerl et al., 1995). It is now widely believed that the deposition of Aβ is an early initiator of neurodegeneration in AD, thus finding methods that can reduce Aβ or enhance its clearance could be a strong therapeutic target. In this sense, autophagy appears to be the first line of defense against accumulation of Aβ.

### Assessing Aβ Pathology in CSF Samples

Cerebrospinal fluid Aβ40 and Aβ42 may be useful (in addition to other biomarkers) in assessing efficacy of drugs such as BACE1 inhibitors, which selectively decrease toxic forms of Aβ (Kennedy et al., 2016). For instance, when CSF levels of Aβ42 are still rising during early stages of amyloid pathology, decreased levels of CSF Aβ42 measured after treatment would show a successful outcome, but at a later stage in amyloid pathology the same finding may indicate accelerated plaque formation (Liu et al., 2004). Anti-amyloid agents are most likely more effective during early AD since deposition of the protein begins many years before diagnosis (Musiek and Holtzman, 2015). Promoting the elimination of Aβ by enzymatic degradation or by clearance enhancement may halt both the aggregation and the accumulation of the peptide (Menendez-Gonzalez et al., 2018). The choroid plexus is known to produce Aβ (Krzyzanowska and Carro, 2012), thus the effect of Aβ synthesis inhibitors on CSF Aβ is bound to reflect changes both sourced in the brain and in the choroid plexus. In addition, evidence suggests that the choroid plexus can remove substances, such as Aβ, from the CSF (Matsumoto et al., 2015).

#### BACE1 Inhibitors

A potent BACE1 inhibitor known as Verubecestat has been shown to reduce plasma, CSF and brain levels of Aβ40, Aβ42, and soluble APPβ (a direct product of BACE1 enzymatic activity) after short- and long-term administration in rats and monkeys (Kennedy et al., 2016). Recently, a study in healthy elderly AD subjects who received treatment with a BACE1 inhibitor showed no alterations in CSF BACE1 levels following treatment, but revealed a strong link between levels of CSF BACE1 and downstream markers such as CSF Aβ42 (Timmers et al., 2017). Genetic deletion of BACE1 eliminated Aβ production and resolved the amyloid plaques and cognitive deficits observed in transgenic mice over-expressing human APP with familial AD mutations (Dominguez et al., 2005; McConlogue et al., 2007; Ohno et al., 2007). Researchers have demonstrated that long-term BACE1 inhibition diminishes CSF tau levels both in early depositing APP transgenic mice and APP transgenic mice with moderate Aβ pathology (Schelle et al., 2017). Overall, BACE1 inhibition appears to not only reduce Aβ generation, but also downstream AD neuropathology (Schelle et al., 2017).

#### γ-Secretase Inhibitors

γ-secretase inhibitors have also proven promising as a therapeutic approach; APP/PS1 mice treated with this compound displayed that a modest decrease (∼30%) of Aβ in ISF was enough to halt amyloid plaque development (Yan et al., 2009). Also, NGP 555 (a γ-secretase inhibitor) has been shown to shift amyloid peptide production to the smaller, non-aggregating forms of amyloid (Olsson et al., 2014; Kounnas et al., 2017). Inhibition of γ-secretase has initially been unsuccessful as a therapeutic target (Cummings, 2010), but more recent compounds have been shown to avoid notch-related toxicity and side effects (Basi et al., 2010; Imbimbo et al., 2010). One failure of slowing Aβ production in patients may be that non-homogenous groups of patients have been included in the trials, and that the treatment has been administered too late in the disease course or has been too short (Bjerke and Engelborghs, 2018). In line with this, some clinical trials have reported changes in CSF Aβ42, but no improvement in clinical endpoints (Ritter and Cummings, 2015).

## Comparative Biomarking

Studies aiming to translate findings between AD system models and patients found that tau derived from AD brains injected into susceptible mouse models induced prion-like tau aggregation (Skachokova et al., 2019). CSF from AD or MCI patients injected into the hippocampus of young P301S tau transgenic mice increased tau phosphorylation and NFT formation 4 months following injection. Post-seeding, the injections accentuated tau pathology in the contralateral hippocampus of the mice, indicative of spreading (Skachokova et al., 2019). Other researchers found that peritoneal dialysis reduced plasma Aβ levels in both chronic kidney disease patients and APP/PS1 mice. ISF Aβ levels in APP/PS1 mice immediately decreased after reducing blood Aβ by peritoneal dialysis. The treatment also attenuated other AD-type pathologies, including inflammation, tau hyperphosphorylation, neurodegeneration, synaptic dysfunction, and rescued the behavioral deficits of the mice. Importantly, the Aβ phagocytic function of microglia was enhanced in APP/PS1 mice after peritoneal dialysis (Jin et al., 2017). Current strategies for clearing Aβ focus on introducing agents into the brain (Jin et al., 2017), but this likely causes adverse effects such as neuroinflammation and tissue scarring (Iijima-Ando et al., 2008; Liu et al., 2012), in addition to increased endogenous tau levels.

### The ABC Scoring System

Well-characterized mouse models hold great translational value given that identifying patients at preclinical AD stages has proven difficult (Bacioglu et al., 2016). However, there are important differences between species, which should be kept in mind while interpreting results (Barten et al., 2017). The ideal translational model for AD would require Aβ and tau deposition in a pathological manner and disease-relevant accumulation of amyloid plaques and tangles similar to that seen in AD patients (Keene et al., 2016). The ABC scoring system (Table 2) can be used to determine the level of AD neuropathological change in both system models and patients. The ABC score is generated by a summary of measures of amyloid plaque distribution A0 to A3 (Thal stages), NFT distribution B0 to B3 (Braak stages), and cortical neuritic plaque density C0 to C3 [Consortium to Establish a Registry for Alzheimer’s Disease (CERAD) score] (Keene et al., 2016). The ideal translational model system of human AD would display amyloid plaques and NFTs in a spatial and temporal manner correlating with “no” or “low” AD pathology at early ages, progressing to “intermediate” and “high” AD pathology at older ages or in the presence of gene mutations related to neuropathological development (Keene et al., 2016).

**TABLE 2:** The ABC scoring system developed by NIA-AA.

**Assessment**	**NIA-AA scoring**
Aβ plaques	A0 (not)
	A1 (low)
	A2 (intermediate)
	A3 (high)
NFTs, including pretangles and threads	B0 (not)
	B1 (low)
	B2 (intermediate)
	B3 (high)
Neuritic and diffuse plaque density	C0 (not)
	C1 (sparse)
	C2 (moderate)
	C3 (frequent)

*The scoring system can be used to help assess and validate the neuropathological features of AD in mouse lines that are potential preclinical models for AD research. Reproduced with permission from Keene et al. (2016). NIA-AA, Alzheimer’s Association; Aβ, amyloid-β; NFT, neurofibrillary tangle.*

In terms of the specifics of the ABC scoring system (Table 2), scoring of diffuse Aβ plaques is based on assessment in the cerebral cortex, hippocampus, striatum, midbrain, brainstem, and cerebellum (Box 1) according to staging established by Thal et al. (2002) resulting in a Thal phase 0–5, which is translated into the NIA-AA score of A0-A3. Meanwhile, scores for NFTs are determined in the *trans-*entorhinal cortex, corpora ammonis, fronto-parietal cortex, and primary visual cortex (Box 1) to generate a Braak stage (Braak and Braak, 1991), which is translated into the NIA-AA score of B0-B3. Since most existing mouse models do not generate NFTs, the NIH have developed a modified B score for p-tau pathology, including distribution of pre-tangles and threads. In addition, most existing mouse models do not form neuritic plaques (contains fibrillar Aβ), so a C score for CERAD (Sperling et al., 2011) neuritic plaque density (none, sparse, moderate, or frequent) and a modified C score for diffuse plaque (does not contain fibrillar Aβ) density are generated from frontal and parietal cortex. Considering that behavioral data from mice have replication issues and are challenging to translate to patients, memory testing *per se* should not constitute a validation criterion or a drug testing endpoint (Keene et al., 2016).

### CSF Collection Methods

When translating CSF biomarkers between system models and patients, an advantage with subcutaneous access systems is that drugs can be dosed and also samples can be obtained from unanesthetized animals, as is typical with humans, and without the confound of anesthesia on CSF production or flow. It has been shown that anesthesia can cause disturbances in neurotransmitter density and cell metabolism, and therefore most times, it is desirable to perform experiments on non-anesthetized animals (Kehr, 1999). It is important to note that in most rodent studies, CSF is collected from the cisterna magna above the spinal column, whereas in humans most CSF is collected from the lumbar spinal vertebrae, and therefore drainage from this latter area in preclinical models has a translational advantage (Barten et al., 2017).

Due to the difficulties in collecting CSF samples from preclinical models the quality of the sample may be comprised, and therefore should be tested. The quality of the sample is most often affected by blood and brain-derived protein contamination (Barten et al., 2017). It is critical to minimize blood-contamination when analytes of interest are found in much higher concentration in the blood compared to the CSF, such as the 1:50,000-fold gradient of tau. Another major contamination source of CSF is proteins released in the brain during the collection procedure; this has a higher impact on tau levels compared to Aβ (Barten et al., 2005).

Furthermore, biomarker development for clinical utility is currently being hampered as comparisons of measurements and techniques between laboratories tend to be unreliable. Factors that may induce variability include storage in different tube types, different aliquot volumes, and the number of freeze-thaw cycles performed, which significantly influences CSF biomarker concentrations (Clough, 2005; Bjerke and Engelborghs, 2018). For instance, CSF Aβ42 measures have been found to be greatly influenced by pre-analytical factors such as the type of collection tube used and the number of freeze-thaw cycles of the sample (Perret-Liaudet et al., 2012b; Toombs et al., 2013; Leitão et al., 2015). Various studies have determined the importance of tube types when collecting CSF samples, highlighting that it is crucial to use polypropylene vials (Perret-Liaudet et al., 2012a), and that the tubes are filled to enhance the volume to surface ratio (Perret-Liaudet et al., 2012a). Ultimately there is poor standardization of biobanking protocols and assay consistency, a fact that hampers novel biomarker discoveries and replication of important findings (Teunissen et al., 2018).

One of the reasons why non-human primates are being preferred over canine or rodent species in CSF studies is that they share an upright orientation of the spinal column akin to humans (Spector et al., 2015). Moreover, the difference in brain size (and thereby CSF volume) between mice and humans is over 3000 times, whereas macaque brains are 10- to 20-fold smaller than human brains. Therefore, the distance from the CSF compartments to deeper regions of the brain significantly varies across species and likely influences the exchange analytes (Spector et al., 2015). Nevertheless, most differences across species are otherwise minor, including the volume ratio of CSF to the brain, ranging from 9 to 18% across species (Barten et al., 2017). CSF turnover per day is also similar between humans and macaques, but is approximately 2-fold higher for rats and 3-fold higher for mice (Barten et al., 2017). The higher turnover rate for rodents can be explained by movement of CSF initiated by the ventricles and arterial pressure as a result of a fast heartbeat, which creates an increase in back- and-forth movement of the CSF (Feinberg and Mark, 1987; Sweetman and Linninger, 2011).

### The Physiology of CSF

The exchange of CSF analytes (including therapeutics) may be highly different between patients and rodents as the latter have a much faster heart rate. However, this factor has not been studied. Non-human primates are most likely better preclinical *in vivo* models for biomarker translation because body weight-based allometric scaling is comparable (Karelina et al., 2017). Moreover, ventricular CSF from AD patients has been found to contain a rare high-molecular-weight tau species that was found to exert high seeding activity (Takeda et al., 2016). This needs to be kept in mind when comparing CSF samples gathered from different regions. Moreover, the sleep-wake cycle regulates ISF and CSF levels of Aβ in AD (Holth et al., 2019), and it has been shown that chronic sleep deprivation increases Aβ plaques. Mouse ISF tau was found to increase ∼90% during normal wakefulness versus sleep and ∼100% during sleep deprivation. The relevant study found that sleep deprivation significantly increased CSF Aβ by 30% (Lucey et al., 2018). This means that the time of day is bound to influence Aβ levels in CSF, which suggests that the timing of sampling needs to be chosen with care and kept consistent between clinical sampling and experiments for correct comparisons.

### The Efficacy of Drugs

One major drawback of comparative biomarking is the translatability of AD drugs from animal models to human clinical trials. Compared to preclinical models, AD pathology in patients develops over decades rather than over months. Longitudinal studies in AD system models may determine the initiation and progression of biomarkers that allow for evaluation of disease-modifying drugs. For example, research shows that BACE1 inhibitors can decrease both plasma and CSF Aβ40 and Aβ42 concentrations in mice, guinea pigs (Tagawa et al., 1991), and non-human primates (Sankaranarayanan et al., 2009; Gravenfors et al., 2012; Jeppsson et al., 2012; Wu et al., 2012). A separate study found that a γ-secretase inhibitor reduced CSF Aβ production in rhesus monkeys without a subsequent rise in Aβ production (Cook et al., 2010). Candidates for therapeutics could be further addressed by extending these findings to translational transgenic AD models and may ultimately offer insights into mechanisms of the disease. Furthermore, increased plasma levels of interleukin-10 has been shown following Aβ immunotherapy in Tg2576 mice (Town et al., 2002; Kim et al., 2007). In a separate study, researchers found that peripheral structures play important roles in clearing Aβ sourced from the brain, suggesting that removing Aβ from the blood may also be effective as an AD therapy (Jin et al., 2017).

γ-secretase modulators have proven especially useful as therapeutic candidates because they do not alter the total amount of Aβ peptides produced by γ-secretase activity, instead, they spare the products of other γ-secretase processing, such as notch (Toyn et al., 2016). Importantly, these compounds do not accelerate the production of the potentially toxic product BACE1-C-terminal fragment (C99) (Toyn et al., 2016). In all species, research suggests that γ-secretase modulator treatment decrease Aβ42 and Aβ40 levels while increasing Aβ38 and Aβ37 by a corresponding amount. Therefore, the mechanism of action of γ-secretase modulators may translate well across species, validating its therapeutic strategy for utility in AD (Toyn et al., 2016).

Other translational research across species has shown increased levels of plasma interleukin-10 following Aβ immunotherapy in Tg2576 mice (Town et al., 2002; Kim et al., 2007). Thereby, translation of inflammatory mechanisms and their peripheral markers may benefit from investigation of changes in microglial markers. However, when interpreting and comparing immune markers from mice caution is warranted, as recent evidence suggests that these markers do not translate well to human inflammatory diseases (Seok et al., 2013). Moreover, increased levels of isoprostanes have been shown in Tg2576 mice prior to plaque formation (Pratico et al., 2001), suggesting isoprostane levels may be useful as a predictive biomarker. Neurofilament light in bodily fluid constitutes a biomarker of neurodegeneration reflecting its translational value in system models and in clinical settings (Bacioglu et al., 2016).

### Comparative AD Neuropathology

The earliest (current) detectable Aβ deposition in humans is the formation of diffuse plaques, whereas in the brain of Tg2576 mice diffuse plaques are not observed until 12 months of age, which is 4 months after biochemically detectable alterations of Aβ (Kawarabayashi et al., 2001). The most common observation in AD patients is minor amounts of Aβ40 deposited in the brain, whereas in 33% of patients great amounts of this Aβ variant are detected. Intriguingly, this latter group of patients also display substantial amyloid angiopathy (amyloid build up on the walls of the arteries in the brain) (Gravina et al., 1995). Similarly, the Tg2576 mouse model displays marked angiopathy and the deposition of a large amount of Aβ40 (Gravina et al., 1995). Generally CSF Aβ40 levels are much higher in patients compared to mice, while brain concentrations are similar (Karelina et al., 2017). In terms of plasma concentrations of Aβ40, this is highly similar between patients and mice, and therefore the greater Aβ40 concentrations observed in human CSF may likely reflect a higher brain production of the peptide (Karelina et al., 2017).

**FIGURE 4:**
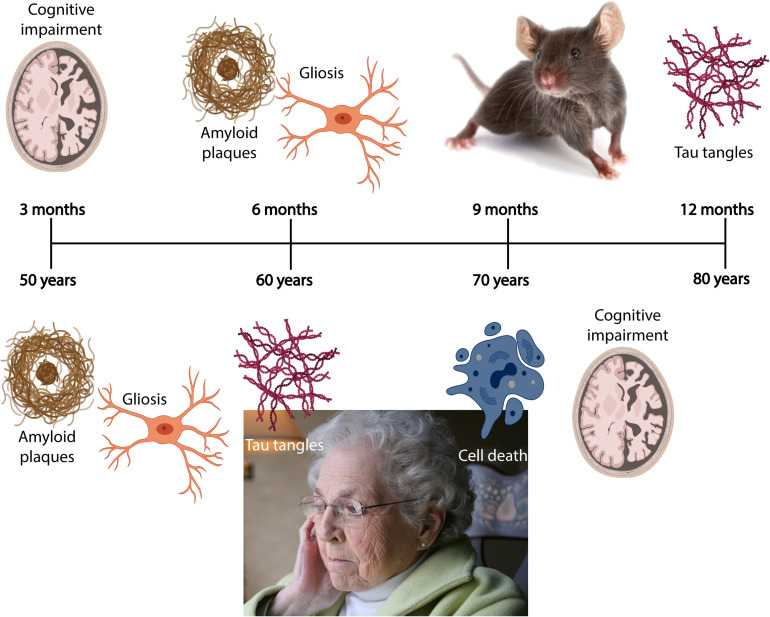
A comparison of pathology and subsequent symptoms along the AD disease cascade between a preclinical model and patients. In the 3xTg AD mouse model (Oddo et al., 2003), cognitive impairment is observed at 3 months of age, whereas amyloid plaques and gliosis are present at 6 months of age. Tau pathology is first present at 12 months of age. In the typical sporadic AD patient, amyloid plaques and associated gliosis may be abundant at 50 years of age, and this pathology is followed by NFTs at approximately 60 years of age. Between 70 and 80 years of age, neurodegeneration occurs, and cognitive impairment becomes prominent in patients. AD, Alzheimer’s disease; NFTs, neurofibrillary tangles. Images were generated using BioRender or taken from a public database.

In terms of the similarity between system models and patients, and specifically transgenic mice and human patients, mice and humans share virtually the same set of genes. Almost every gene found in mice or humans has been observed in a closely related form in the other. To look directly at differences along the AD disease cascade, we compare pathological events between the 3xTg AD mouse model and sporadic AD patients (Figure 4). The 3xTg AD mouse model develops amyloid and tau pathology, including amyloid plaques and NFTs (Oddo et al., 2003). At 3 months of age these mice have developed cognitive impairment (Oddo et al., 2003), whereas at approximately 50 years of age an AD patient has developed amyloid plaques and gliosis (Braak and Del Trecidi, 2015). At 6 months of age, the mice develop amyloid plaques and gliosis (Oddo et al., 2003), while patients develop tau tangles at approximately 60 years of age (Braak and Del Trecidi, 2015). At 70 years of age, patients usually suffer from neurodegeneration because of the presence of tau tangles and exhibit cognitive impairment (Braak and Del Trecidi, 2015). First at 12 months of age will the mice develop tau tangles (Oddo et al., 2003).

There are two important differences observed along the AD disease cascade between the species: first, mice exhibit cognitive impairment prior to tau tangles, whereas cognitive impairment is most likely a result from neurodegeneration caused by NFTs in human patients. Second, one does not observe neuronal death in this mouse model, while neuronal death is thought to be the sole causative pathology for symptom development in AD patients. In line with this, by comparing CSF, plasma and *in vivo* amyloid imaging, cross-sectional data obtained at baseline in individuals from AD families enrolled in the Dominantly Inherited Alzheimer Network (DIAN) show lower concentrations of CSF Aβ42 when amyloid plaques accumulate, and elevated concentrations of CSF t-tau and p-tau in mutation carriers 10–20 years prior to symptom onset and detection of cognitive deficits (Fagan et al., 2014). This highlights the need for longitudinal CSF sampling in animals modeling AD, in order to compare biochemical, imaging and behavioral tests against each other, and eventually to patients. However, given the difficulty of identifying patients at preclinical AD stages, it is important to remember that well-characterized preclinical disease models hold great translational value (Mattsson et al., 2017a).

## Conclusion and Future Directions

Clearly, there is a pressing need for better quality data from model systems investigating biomarkers that can be directly translated to human biomarkers. The core biomarkers (Aβ42, t-tau, and p-tau) have been found to translate well across species, whereas biomarkers of inflammation translate to a lesser extent between mouse models and patients. Researchers should use autophagic and synaptic degeneration markers when analyzing samples from preclinical models because these markers appear promising in predicting development of AD. Changes in levels of autophagic markers and neurofilament light correlate strongly with the core biomarkers of AD, and other novel biomarkers should be tested in combination in preclinical models to validate findings observed in patients. Currently, non-invasive structural and functional imaging can detect AD onset and longitudinally monitor disease progression in AD patients. By the combination of early predictive CSF biomarkers, imaging modalities can be strengthened in their ability to characterize patients along the disease cascade. Additional non-invasive methods for detecting AD biomarkers need to be established, such as blood sampling, which could be used in combination with CSF sampling. CSF sampling is invasive but reflects changes in protein levels in the brain to a greater extent compared to blood-based markers. Another advantage of CSF sampling compared to blood testing is that Aβ is found in the periphery, so it is difficult to differentiate between brain- and periphery-based Aβ levels. Additionally, there are greater levels of tau in ISF compared to CSF, so this needs to be controlled for when analyzing CSF samples. Neurofilament light is transferable between CSF and plasma in humans, but this needs to be verified in system models.

Furthermore, large inter- and intra-laboratory variations in biomarker sampling may have great consequences in terms of comparisons of results, while within individual laboratories such variations may affect planning and interpretations of longitudinal studies (Blennow et al., 2015). One also needs to consider differences in the CSF sampling methods used, given that protein levels differ between ventricular and lumbar CSF. Studies have shown that changes in the levels of core biomarkers as measured in CSF may be useful for assessing the efficacy of drugs. When translating findings across species, it is important to use the same scoring or grouping system when assessing changes in biomarkers and observed neuropathology. Ultimately, an early diagnosis by utilizing biomarkers detectable at this stage of disease will be the cornerstone for early identification of patients that are regressing away from the prodromal stage of AD (Teunissen et al., 2018). There is a need for continued progress within the AD biomarker field so that markers can be translated across animal models and clinical populations to serve as a translational bridge between model systems and clinical populations (Sabbagh et al., 2013). We still cannot translate all pathological hallmarks seen in AD patients to preclinical models, and we therefore need to be aware of pertinent differences when comparing AD research across species and bringing findings into the clinic.

## Author Contributions

All authors contributed to the content of the article, and critically reviewed and edited the manuscript.

## Conflict of Interest

The authors declare that the research was conducted in the absence of any commercial or financial relationships that could be construed as a potential conflict of interest.
